# Developing a *Nicotiana benthamiana* transgenic platform for high‐value diterpene production and candidate gene evaluation

**DOI:** 10.1111/pbi.13574

**Published:** 2021-03-18

**Authors:** Edith C.F. Forestier, Tomasz Czechowski, Amy C. Cording, Alison D. Gilday, Andrew J. King, Geoffrey D. Brown, Ian A. Graham

**Affiliations:** ^1^ Centre for Novel Agricultural Products Department of Biology University of York Heslington York UK; ^2^ Department of Chemistry University of Reading Reading UK

**Keywords:** casbene, double bond reductase, *Euphorbiaceae*, *Jatropha curcas*, jolkinol, lathyranes

## Abstract

To engineer *Nicotiana benthamiana* to produce novel diterpenoids, we first aimed to increase production of the diterpenoid precursor geranylgeranyl pyrophosphate (GGPP) by up‐regulation of key genes of the non‐mevalonate (MEP) pathway sourced from *Arabidopsis thaliana*. We used transient expression to evaluate combinations of the eight MEP pathway genes plus GGPP synthase and a *Jatropha curcas* casbene synthase (*JcCAS*) to identify an optimal combination for production of casbene from GGPP. *AtDXS* and *AtHDR* together with *AtGGPPS* and *JcCAS* gave a 410% increase in casbene production compared to transient expression of *JcCAS* alone. This combination was cloned into a single construct using the MoClo toolkit, and stably integrated into the *N. benthamiana* genome. We also created multigene constructs for stable transformation of two *J. curcas* cytochrome P450 genes, *JcCYP726A20* and *JcCYP71D495* that produce the more complex diterpenoid jolkinol C from casbene when expressed transiently with *JcCAS* in *N. benthamiana*. Stable transformation of *JcCYP726A20*, *JcCYP71D495* and *JcCAS* did not produce any detectable jolkinol C until these genes were co‐transformed with the optimal set of precursor‐pathway genes. One such stable homozygous line was used to evaluate by transient expression the involvement of an ‘alkenal reductase’‐like family of four genes in the further conversion of jolkinol C, leading to the demonstration that one of these performs reduction of the 12,13‐double bond in jolkinol C. This work highlights the need to optimize precursor supply for production of complex diterpenoids in stable transformants and the value of such lines for novel gene discovery.

## Introduction

Plant diterpenes containing a *gem*‐dimethylcyclopropane subunit, mostly found in species of the *Euphorbiaceaea*, are of much interest across various industrial sectors including pharmaceuticals due to their bioactivity and structural complexity (Durán‐Peña *et al*., 2014). However, the low abundance in the natural host and difficulties in chemical synthesis owing to high structural complexity often limit the development of industrial applications for these compounds (Andersen‐Ranberg et al., 2016). Ingenol mebutate from *Euphorbia peplus* and tigilanol tiglate from *Fontainea picrosperma* are examples of casbene‐derived *gem*‐dimethylcyclopropane diterpenes that exemplify the fact that even when these compounds are developed as products, the supply chain remains challenging for industry. Ingenol mebutate is a licensed treatment for actinic keratosis (Picato|European Medicines Agency), that is sourced either from ingenol semi‐synthesis (Liang *et al*., 2012) or by direct extraction from the plant, yielding no more than 1.1 mg/kg (Hohmann *et al*., 2000). Tigilanol tiglate is an experimental drug already approved for treating dog tumours mast cells (Ridder *et al*., 2020) but obtained solely from the extraction from seeds of *Fontainea picrosperma*, a sub‐canopy tree from a restricted area of Queensland rainforest (Lamont *et al*., 2016). Developing new sustainable production platforms for high value diterpenoids would improve the supply chains of existing diterpene‐derived drugs and provide the confidence needed to exploit the huge potential that this class of compounds has to offer.

*Nicotiana benthamiana* represents a well‐established heterologous expression system to address this issue. Transient foreign gene expression mediated by *Agrobacterium tumefaciens* infiltration using either syringe or vacuum infiltration (Kapila *et al*., 1997; Reed *et al*., 2017) results in production of recombinant proteins or metabolites (Hasan *et al*., 2014; McCormick *et al*., 1999; Whaley *et al*., 2011). Companies such as Leaf Expression System (Norwich, UK) or Kentucky BioProcessing Inc (Owensboro, KY, USA) have scaled‐up this *N. benthamiana* platform for production of antibodies, antigens and enzymes (https://kentuckybioprocessing.com, https://www.leafexpressionsystems.com).

Transient gene expression in *N. benthamiana* is a routine and valuable tool for functional characterization of genes involved in plant metabolism including diterpenoids (Andersen‐Ranberg et al., 2016; King *et al*., 2014, 2016; Reed and Osbourne, 2018), but scaling up the approach to produce significant amounts of end product is not routine, with one exception being the production of the triterpene β‐amyrin at mg/g leaf DW amounts (Reed *et al*., 2017; Stephenson *et al*., 2018). Vacuum infiltration requires substantial upstream work such as growing large volumes of *A. tumefaciens* and this can become more of a limiting factor when multiple genes need to be expressed as is the case for production of end products of complex metabolic pathways.

We reasoned that stable transformation of *N. benthamiana* to produce either a valuable end product or a key intermediate would simplify the production process as once stable lines are obtained they could be maintained as seeds and grown at scale. We targeted production of jolkinol C, a member of the lathyrane class of casbene‐derived diterpenes and a presumed intermediate of both ingenol mebutate and tigilanol tiglate described above (King *et al*., 2014, 2016; Luo *et al*., 2016). The production of such stable transformants producing intermediates in complex biochemical pathways could also possibly serve as a valuable tool for functional characterization by transient expression of candidate genes associated with the latter stages of such pathways.

**Figure 1:**
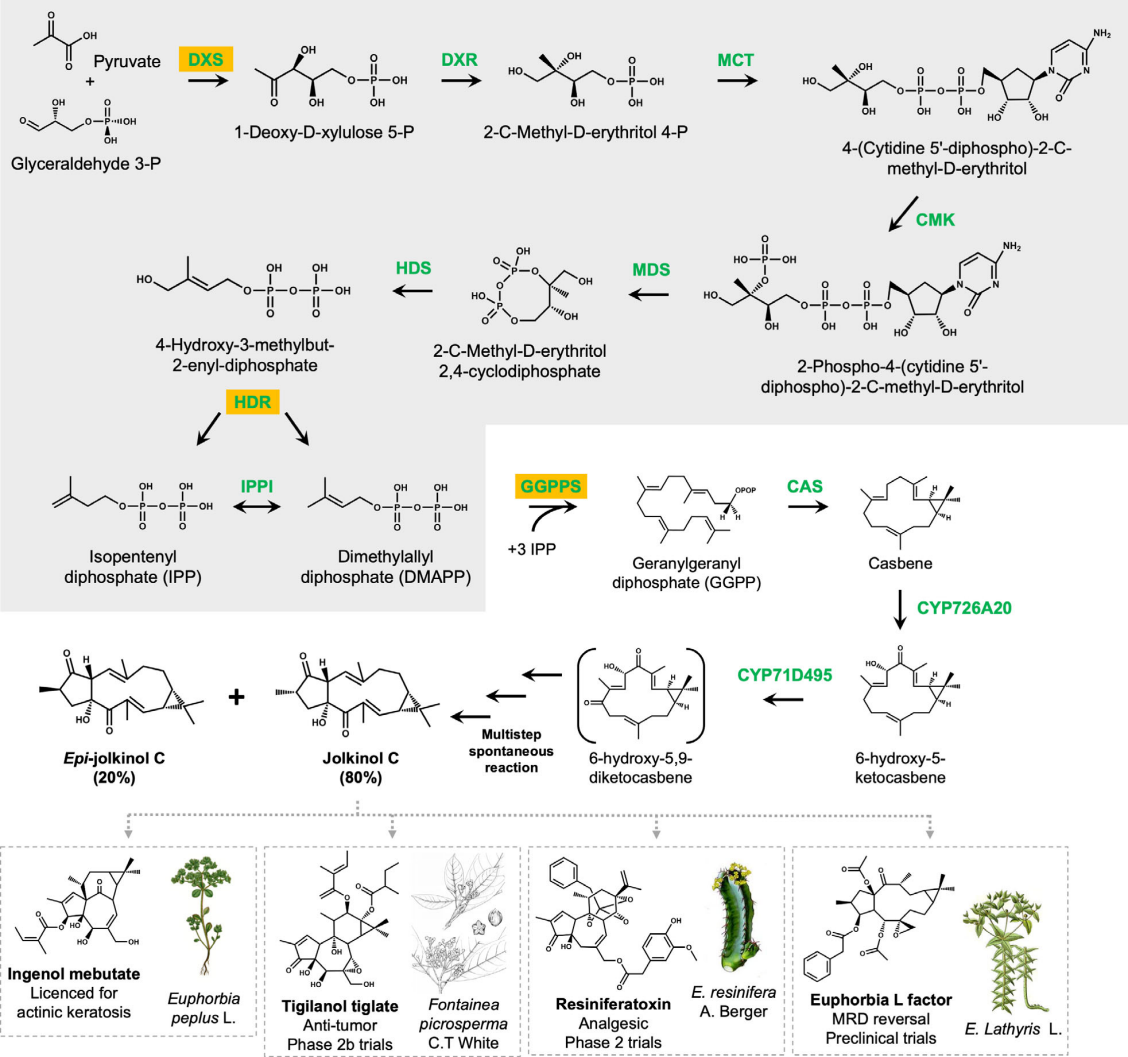
Biosynthetic steps involved in production of jolkinol C and *epi*‐jolkinol C from pyruvate and glyceraldehyde 3‐P *Euphorbia* species (modified from King *et al*., 2016). The MEP pathway (shaded) provides precursors for diterpene production. Jolkinol C is a potential intermediate in the production of a diverse range of bioactive casbene‐derived diterpenoids (lower panel). MEP pathway enzymes are DXS (1‐deoxy‐D‐xylulose 5‐phosphate synthase), DXR (1‐deoxy‐D‐xylulose 5‐phosphate reductoisomerase), MCT (2‐C‐methyl‐D‐erythritol 4‐phosphate cytidylyltransferase), CMK (4‐(cytidine 5’diphospho)‐2‐*C*‐methyl‐D‐erythritol kinase), MDS (2‐C‐methyl‐D‐erythritol 2,4‐cyclodiphosphate synthase), HDS (4‐hydroxy‐3‐methylbut‐2‐enyl‐diphosphate synthase), HDR (4‐hydroxy‐3‐methylbut‐2‐enyl diphosphate reductase) and IPPI (isopentenyl diphosphate Δ‐isomerase). The highlighted enzymes have been shown to influence production of GGPP (Brückner and Tissier, 2013; Botella‐Pavía *et al*., 2004). Note: 6‐hydroxy‐5,9‐diketocasbene undergoes a series of spontaneous reactions resulting in jolkinol C and epi‐jolkinol C (King *et al*., 2016).

Our engineering approach aimed to (i) optimize the flux of carbon from pyruvate and glyceraldehyde 3‐P of primary metabolism through the MEP pathway to the diterpene precursor geranylgeranyl pyrophosphate (GGPP) (Gershenzon and Croteau, 2018) and (ii) combine this with addition of a casbene synthase (CAS) which cyclizes GGPP into casbene (Dueber *et al*., 1978) and two cytochrome P450s that oxidize casbene to produce an intermediate that undergoes non‐enzymatic ring closure to produce jolkinol C (King *et al*., 2016; Figure 1). We initially used transient expression to identify enzymatic steps of the MEP pathway that would increase GGPP as determined by production of casbene yield, then combined this novel combination of genes with the casbene oxidizing enzymes. This work reports on the successful production of jolkinol C in stable homozygous transformants of *N. benthamiana* and how we then exploited these to determine the function of a novel jolkinol C modifying enzyme.

## Results and discussion

### Transient expression in *Nicotiana benthamiana* to determine the optimal combination of MEP pathway genes for production of casbene

Previous reports have shown that DXS, the first committed enzyme of the MEP pathway, is critical in the synthesis of IPP and DMAPP in many plants (Estévez *et al*., 2001; Gong *et al*., 2006; Lois *et al*., 2000; Morris, 2006). For example, when the diterpene synthase cembratrien‐ol synthase was expressed in combination with DXS and GGPPS, there was a significant increase in the production of the diterpene, while the over‐expression of GGPPS alone was inconclusive (Brückner and Tissier, 2013). The enzyme 4‐hydroxy‐3‐methylbut‐2‐enyl diphosphate reductase (HDR) has also been suggested to play a notable role in controlling the production of MEP‐derived precursors (Botella‐Pavía *et al*., 2004). Its over‐expression in *Arabidopsis thaliana*, together with a taxadiene synthase, led to a 13‐fold increase of taxadiene level compared to diterpene synthase expressed on its own. We therefore decided to test combinations of *DXS* and *GGPPS* with *HDR* and the remaining MEP pathway genes (*DXR*, *MCT*, *CMK*, *MDS*, *HDS* and *IPPI*; Figure 1) to establish whether we could further increase the flux towards production of diterpenoid precursors. MEP pathway genes from *Arabidopsis thaliana* (Table S1) were cloned under the control of the CAMV35S promoter into the pEAQ‐HT expression vector developed for high expression in *N. benthamiana* (Peyret and Lomonossoff, 2013). In all combinatorial tests, we included a *Jatropha curcas CASBENE SYNTHASE* (*JcCAS*) gene also under control of the CAMV35S promoter in pEAQ‐HT and monitored casbene levels as an indirect measure of GGPP production.

**Figure 2:**
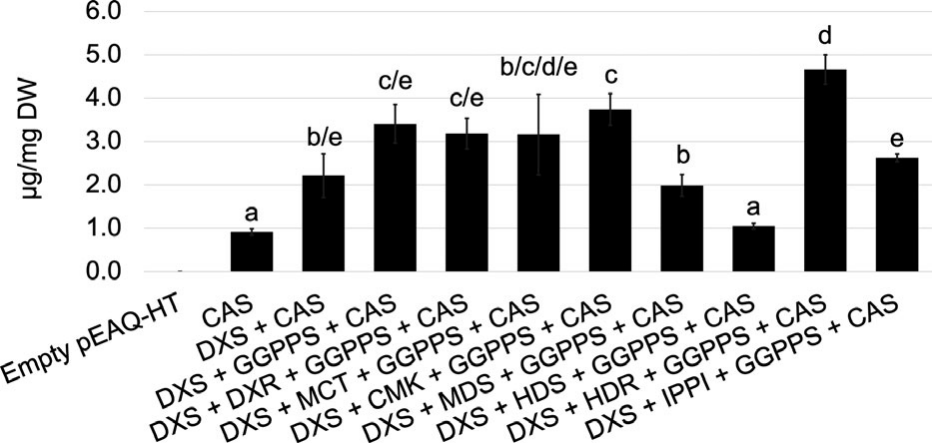
Casbene content in *N. benthamiana* co‐expressing *CAS* and individual MEP pathway genes plus *GGPPS*. Casbene content (µg/mg DW ± standard deviation, *n* = 3). Letters show similarity or significant differences between treatment means (*P* < 0.05, *F*‐test and *T*‐test).

We co‐infiltrated *A. tumefaciens* cultures carrying distinct plasmid constructs to test individual genes and various gene combinations by transient expression. This revealed that of the individual genes, only DXS resulted in an increase in casbene and the optimal combination of MEP pathway genes was *AtDXS* and *AtHDR* together with *AtGGPPS* and *JcCAS* (Figure 2). This combination of four genes gave a 410% increase in casbene production compared to transient expression of *JcCAS* alone (Figure 2), with *AtHDR* contributing 140% of this increase. To the best of our knowledge, this is the first demonstration that this association of these three MEP pathway genes can greatly increase the quantity of GGPP precursor.

Remarkably, addition of either *AtHDS* or *AtMDS* to the *AtDXS*, *AtGGPPS* and *JcCAS* combination resulted in a decrease rather than an increase in casbene production, with levels falling back to those found for expression of the *JcCAS* gene alone after the addition of *AtHDS* (Figure 2). This dominant negative effect of *AtHDS* on casbene production is found in various gene combinations including with the *JcCAS* alone (Figure S1). In *E. coli*, overexpression of *ispG*—encoding the native HDS enzyme—resulted in overproduction of HMBPP, which could cause cytotoxicity by interfering with the synthesis of nucleotides and proteins (Li *et al*., 2017). Activation of the *ispH* gene encoding the *E. coli* HDR enzyme was then able to eliminate the cytotoxic effect of *ispG*. A similar phenomenon may be occurring when *AtHDS* is overexpressed in the *N. benthamiana* transient expression system, as supported by the observation that the dominant negative effect of *AtHDS* on casbene production is removed when the gene is co‐expressed with *AtHDR*, which removes the toxic intermediate and increases the flux towards GGPP production. However, addition of *HDS* to the *DXS* + *HDR* + *GGPPS* combination does not significantly increase the amount of casbene produced and thus we did not include HDS in the optimal combination of MEP pathway genes.

### Development of a single vector multi‐gene system for transient up‐regulation of casbene precursors

Having established the optimal combination of MEP pathway genes for casbene production using separate vectors, we next wanted to express these in a single vector with promoters of moderate strength. The aim was to use these multigene vectors subsequently for stable expression, choosing promoters other than CaMV35S, so as to avoid triggering gene silencing in future stable transgenic lines (Elmayan and Vaucheret, 1996; Mishiba *et al*., 2005). We used the MoClo modular cloning system (Engler *et al*., 2014; Weber *et al*., 2011) as it offers multiple options of expression cassette with different terminator regions and different promoters. We classified the promoters into two groups, A and B, established by Engler and co‐workers on the basis of GFP expression (**Table** **S2** **).** The strength of the promoters was originally described in relation to the amount of GFP fluorescence measured, and this method has been proved to be a quantitative reporter of gene expression (Soboleski *et al*., 2005). Using this criterion, we estimated that promoters of group A were able to provide between 5 and 15% of relative fluorescence compared to the reference construct 35S:GFP. Promoters of group B produced GFP fluorescence of between 25% and 45% as compared to the same reference. Level 2 (L2) MoClo vectors were assembled with distinct promoter and terminator sequences in different gene constructs to avoid homology‐dependent gene silencing when integrated in the genome (Park *et al*., 1996).

**Figure 3:**
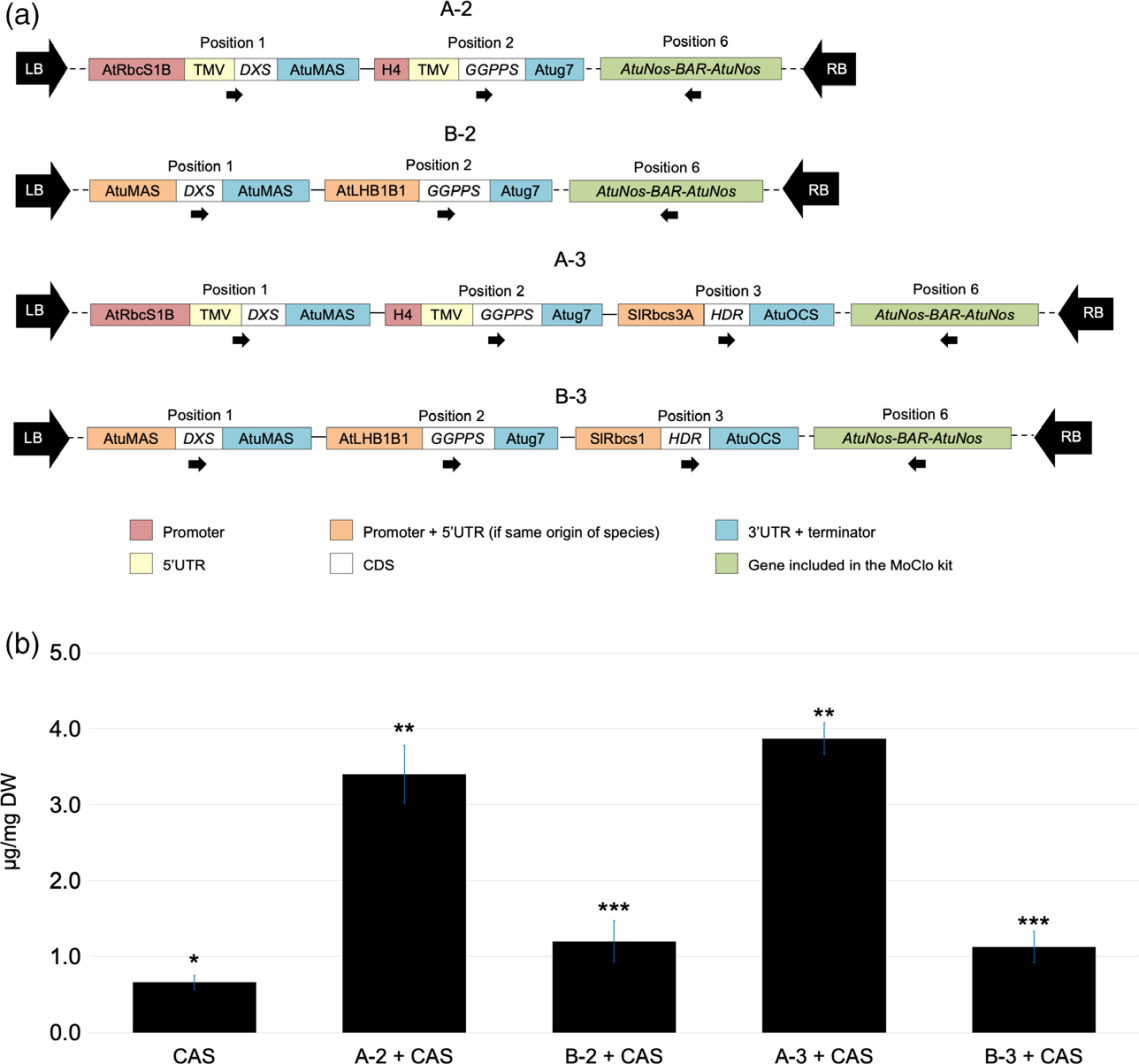
Casbene production in transient expression assays of multigene constructs with different MEP pathway gene combinations and promoter strengths. (a) Multigene constructs obtained by MoClo and their designation. ‘A‐2’ and ‘B‐2’ refer to multigene constructs carrying *AtDXS* and *AtGGPPS* driven by group A or B promoters, respectively. ‘A‐3’ and ‘B‐3’ refer to multigene constructs carrying *AtDXS*, *AtHDR* and *AtGGPPS* driven by group A or B promoters, respectively. All multigene constructs also carry the *Bar* gene in position 6, conferring resistance to the herbicide Basta. *Bar* gene was included in the MoClo kit. (b) Casbene content (µg/mg DW) in *N. benthamiana* transiently expressing *CaMV35S*::*JcCAS* alone or in combination with multigene constructs of MEP pathway genes. In all cases *JcCAS* was inoculated on a separate vector. Casbene content (µg/mg DW ± standard deviation, *n* = 3). Symbols show similarity or significant differences between treatment means (*P* < 0.05, *F*‐test and *T*‐test).

We produced four L2 constructs: two with *AtDXS* and *AtGGPPS* under the control of group A or B promoters (referred to as A‐2 and B‐2) and two with *AtDXS*, *AtGGPPS* and *AtHDR* driven by the same promoter groups (referred to as A‐3 and B‐3; Figure 3a). The *Bar* gene conferring Basta (phosphinothricin) resistance was included in all constructs as they were designed for both transient and stable transformation. Each L2 construct was tested by transient expression in *N. benthamiana* by co‐infiltration with a separate pEAQ‐HT vector containing *35S‐JcCAS* in order to evaluate casbene production when compared with the infiltration of *35S‐JcCAS* alone (Figure 3b). All L2 constructs combined to *35S‐JcCAS* produced significantly more casbene than single infiltration of the latter. Maximum production of casbene of 3.9 µg/mg dry weight was achieved by infiltration of the three gene L2 construct with the group A promoter plus *35S‐JcCAS*. This represents a 485% increase in casbene compared to that produced upon infiltration of the *35S*‐*JcCAS*. We consistently found that both the two gene and three gene L2 constructs under the control of group B promoters gave lower levels of casbene production compared to the group A promoters, suggesting that higher expression is not always better in this transient expression system.

### Engineering stable production of casbene‐derived diterpenoids in *N. benthamiana*

Having demonstrated that we could produce elevated levels of casbene in the transient expression platform, we decided to establish whether a similar result could be achieved by stable transformation of *N. benthamiana* and, if possible, to use this same system for production of casbene‐derived diterpenoids, such as jolkinol C. Previous work in our laboratory had shown that transient expression of *N. benthamiana* with *pEAQ‐HT* vectors containing the individual *J. curcas* genes, *CYP726A20* and *CYP71D495* that encode cytochrome P450 oxidase enzymes, together with pEAQ‐HT::*JcCAS*, enabled the production of jolkinol C and *epi*‐jolkinol C, which are proposed intermediates in the biosynthetic pathways to various bioactive casbene‐derived diterpenoids (Figure 1; King *et al*., 2014, 2016). We used the MoClo modular cloning system to generate gene constructs with group A and B promoter variants of *JcCYP726A20*, *JcCYP71D495 and JcCAS* plus the *NptII* cassette which confers kanamycin resistance in plants. We named these gene constructs A‐CP and B‐CP (“CP” for *CAS*‐*P450s*; Figure S2a). Prior to stable transformation, we evaluated these two constructs by transient expression in *N. benthamiana* and found that they both enabled production of jolkinol C and *epi*‐jolkinol C (Figure S2b). The gene constructs driven by group A promoters produced significantly more jolkinol C than those driven by group B promoters, and more than the combination of genes expressed on separate pEAQ‐HT vectors, which is consistent with the transient expression of the various MEP pathway genes constructs and casbene production.

**Figure 4:**
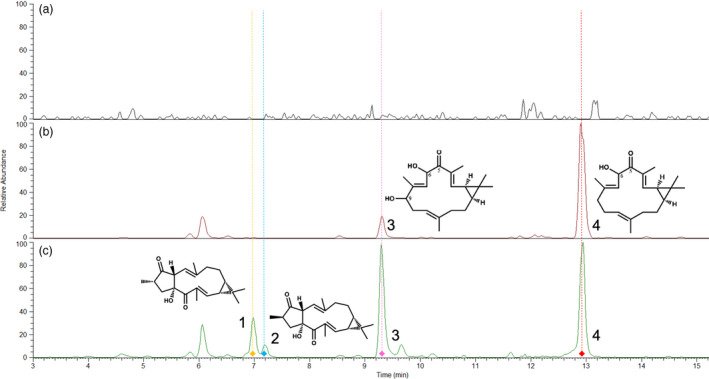
Production of casbene‐derived diterpenes following stable transformation of *N. benthamiana*. (a) Mass chromatogram (UPLC‐MS) of control line carrying resistance markers *Bar* and *NptII* (m/z 299–304). (b) Mass chromatogram of transformant A‐CP n°6 carrying the *JcCYP726A20*, *JcCYP71D495* and *JcCAS* (m/z 299–304). (c) Mass chromatogram of co‐transformant B‐2/A‐CP n°1 carrying *JcCYP726A20*, *JcCYP71D495* and *JcCAS* on one cassette plus *DXS* and *GGPPS* on another (m/z 299–304). 1. Jolkinol C; 2. *Epi*‐jolkinol; 3. 6,9‐dihydroxy‐5‐ketocasbene; 4. 6‐hydroxy‐5‐ketocasbene

Since both A‐CP and B‐CP constructs functioned in transient expression, they were used for stable transformation of *N. benthamiana* using the *A. tumefaciens* leaf disc transformation method (Horsch *et al*., 1989). Single transformants carrying either A‐CP or B‐CP constructs and co‐transformants carrying either A‐CP or B‐CP and one of A‐2, A‐3, B‐2 or B‐3 were produced. To avoid the risk of gene silencing due to identical transgene components, co‐transformations were conducted between A‐2 or A‐3 with B‐CP and B‐2 or B‐3 with A‐CP (Figure S3). Neither jolkinol C nor *epi*‐jolkinol C were detectable in any of the 18 single transformants expressing the *JcCYP726A20*, *JcCYP71D495 and JcCAS*, which contrasts with the results of transient expression. It is noteworthy, however, that an intermediate identified by NMR as 6,9‐dihydroxy‐5‐ketocasbene was detected in the A‐CP single transformants (Figures 4b and Figure S4). This compound differs from the putative direct precursor of jolkinol C, 6‐hydroxy‐5,9‐diketocasbene (King *et al*., 2016), in terms of the extent of oxidation at the C‐9 position. We propose that 6,9‐dihydroxy‐5‐ketocasbene is the product of incomplete oxidation by JcCYP71D495, resulting in a hydroxyl rather than a keto‐group at C‐9. 6,9‐Dihydroxy‐5‐ketocasbene is unable to participate in the same spontaneous aldol reaction forming jolkinol C as 6‐hydroxy‐5,9‐diketocasbene and we therefore propose that this causes it to accumulate in the A‐CP transformants. It is not immediately obvious why JcCYP71D495 should only be catalysing partial oxidation at the C‐9 position, but accumulation of this compound has previously been observed for the same set of genes, when expressed in *S. cerevisiae* (Wong *et al*., 2018). No pathway intermediates or end‐products were detectable in the B‐CP transformants.

Co‐transformation with dual selection on kanamycin and Basta was four times less efficient than single transformation but still resulted in 27 T0 lines, distributed unevenly across the 4 gene vector combinations (Figure S3). Qualitative analyses of jolkinol C and *epi*‐jolkinol C together with their pathway intermediates revealed that 15 of the 27 T0 co‐transformants had detectable amounts of jolkinols (Figure 4c and Table 1). The most consistent combination for jolkinol and *epi*‐jolkinol production was from the B‐2/A‐CP co‐transformant class for which 13 out of 15 primary transformants contained jolkinols. The addition of *HDR* did not result in more jolkinols in the stable lines, but larger numbers of independent transformants would need to be evaluated before concluding that this step is not limiting in provision of the casbene precursor.

**Table 1:** Qualitative detection of oxidized casbene derivatives in the 26 T0 co‐transformant lines and estimation of the copy number of each gene cassette

Condition	T0 line	6H5K	6,9dH5K	*Epi*‐jC	Jolkinol C	Copy number of the different cassette	
						Basta	Kan
A‐2/B‐CP	n°1	−	−	−	−	<1	1
	n°2	−	−	−	−	2	>3
B‐2/A‐CP	n°1	+++	++	+	+	1	1
	n°2	++	+++	+	++	1	3
	n°3	++	+++	+	++	1	1
	n°4	+++	+++	+	+	1	1
	n°5	+++	+++	+	+	1	2
	n°6	++	+++	+	+	Sterile	
	n°7	+	+	−	−	Sterile	
	n°8	+	++	+	+	> 3	1
	n°9	+	++	−	−	1	1
	n°10	+	++	++	+++	< 1	3
	n°11	+	+++	++	+++	< 1	> 3
	n°12	+	+++	++	+++	< 1	1–2
	n°13	+	++	++	+++	< 1	> 2
	n°14	++	++	+	+	< 1	3
	n°15	+	++	++	+++	< 1	2
A‐3/B‐CP	n°1	−	−	−	−	2	1–2
	n°2	−	−	−	−	1	1
	n°3	−	−	−	−	2	2
	n°4	−	−	−	−	1	1
B‐3/A‐CP	n°1	−	−	−	−	1	1
	n°2	−	−	−	−	1	1
	n°3	++	++	−	−	< 1	1
	n°4	−	−	−	−	Sterile	
	n°5	+	+	+	+	1–2	<1
	n°6	+++	+++	+	+	Sterile	

6H5K, 6‐hydroxy‐5‐ketocasbene; 6,9dH5K, 6,9‐dihydroxy‐5‐ketocasbene; epi‐jC, epi‐jolkinol C.

Among the B‐2/A‐CP co‐transformants, the 5 lines showing a relative high content of jolkinol C (symbol +++ in Table 1) displayed morphological abnormalities including narrowed flower corolla and smaller seed pods (Figure S5a); nevertheless, the majority of the transgenic lines produced sufficient quantities of viable seeds to perform segregation analyses.

### Development of a diterpenoid transgenic platform for gene candidate evaluation

We used segregation ratios for the basta and kanamycin selectable marker genes in T1 and T2 progeny of independent transformants to determine copy number and zygosity. This identified three independent transformants (number 1, 3 and 4) that carry a single copy of the B‐2 and A‐CP cassettes (Table 1). We identified the homozygous lines by segregating the T2 generation and analysed the casbene derivatives content (Figure S6). We selected the n°4 B‐2/A‐CP line for its higher content of jolkinol C in the T2, hereinafter referred to as NbJolk‐C, for further analysis and advanced this through to the T3 and T4 generations, at which stage it presented a distinct growth phenotype compared to WT (Figure S5b). Despite slower germination and growth, plus a more upright appearance in the first weeks of development, NbJolk‐C produced leaves, flowers and viable seeds.

**Figure 5:**
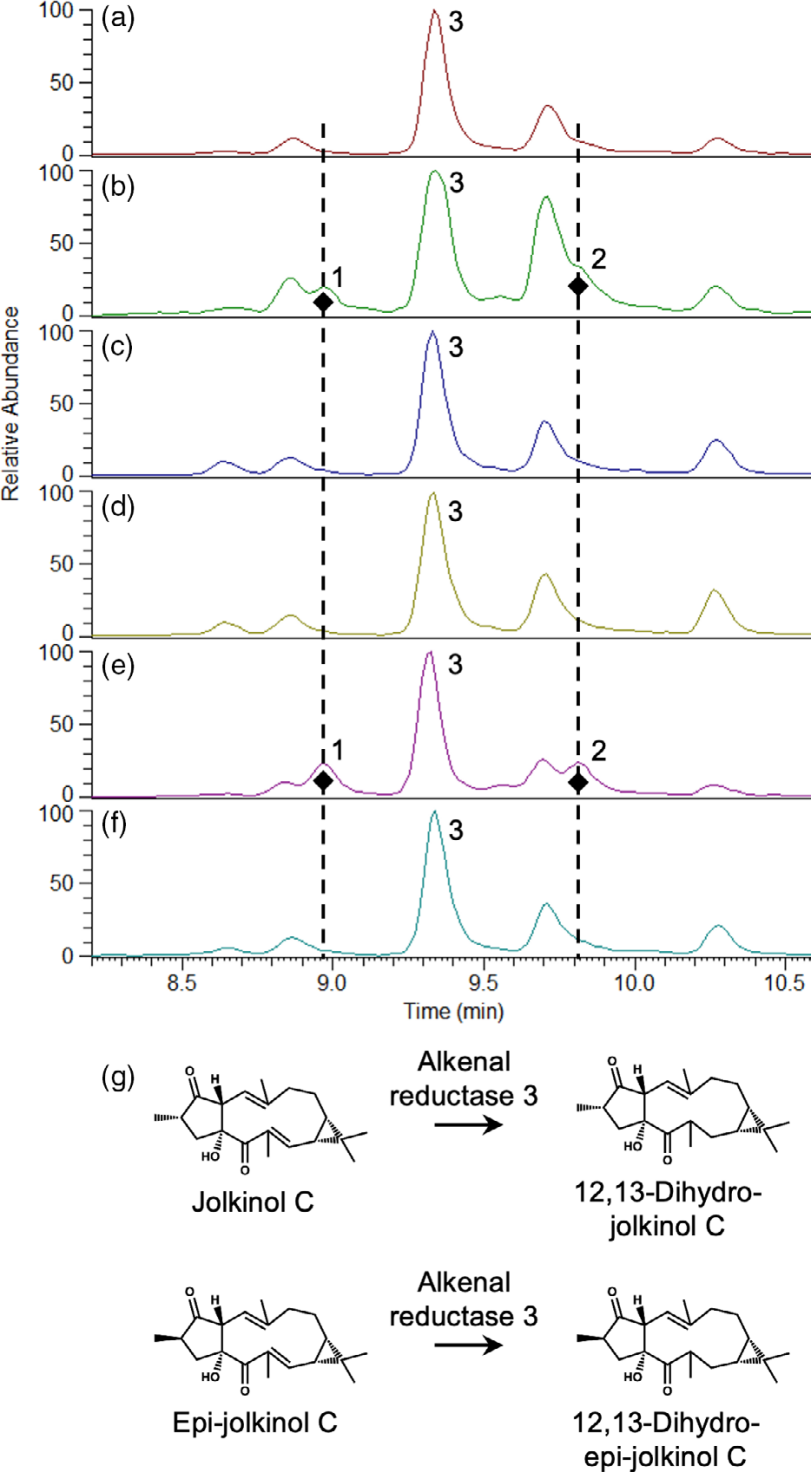
Use of jolkinol C producing platform to evaluate candidate gene function. A stable *N. benthamiana* jolkinol C producing transgenic line was used to assay the function by transient expression of four candidate alkenal reductase genes identified on a *J. curcas* gene cluster (King *et al*., 2016). In all cases, mass spectrometry (UPLC‐MS) is shown at base peak m/z 301. (a) Empty pEAQ‐HT vector control, (b) co‐expression of all four alkenal reductase genes in separate pEAQ‐HT vectors, (c) pEAQ‐HT::alkenal reductase 1, (d) pEAQ‐HT::alkenal reductase 2, (e) pEAQ‐HT::alkenal reductase 3, (f) pEAQ‐HT::alkenal reductase 4 and (g) schematic presentation of the conversion of jolkinol C and epi‐jolkinol C to 12,13‐dihydro‐jolkinol C and 12,13‐dihydro‐epi‐jolkinol C, respectively. 1. 12,13‐dihydro‐jolkinol C; 2. 12,13‐dihydro‐epi‐jolkinol C; 3. 6,9‐dihydroxy‐5‐ketocasbene.

Next we tested whether the T3 and T4 NbJolk‐C material could be used as a transient expression platform to investigate the function of other genes from the *Jatropha curcas* diterpenoid biosynthesis gene cluster that contains *JcCYP726A20*, *JcCYP71D495* essential for production of jolkinol‐C (Figure S7; King *et al*., 2016). We transiently expressed four ‘alkenal reductase’‐like genes from this cluster in combination and individually in NbJolK‐C and discovered that the *alkenal reductase 3‐like* gene results in production of two new compounds (Figure 5). These were identified by NMR spectroscopy as 12,13‐dihydro‐jolkinol C and 12,13‐dihydro‐epi‐jolkinol C (Figure S8), leading us to conclude that the *alkenal reductase 3‐like* gene encodes a double bond reductase enzyme that can reduce the C12‐C13 double bond present in jolkinol C and epi‐jolkinol C.

Biosynthesis of ingenanes, tiglanes and jatrophanes has been proposed in the literature to involve a lathyrane intermediate (Evans and Taylor, 1983; Schmidt, 1987). It is noteworthy that while lathyranes such as jolkinol C contain a C12‐C13 double bond, this position is reduced in these other classes (Durán‐Peña *et al*., 2014; Evans and Taylor, 1983). We can therefore speculate that the activity we report herein for the *J. curcas alkenal reductase 3‐like* gene represents a crucial step in the biosynthesis of these more complex classes of diterpenoids derived from a lathyrane backbone.

## Conclusion

Transient expression, in various combinations, of the entire suite of MEP pathway genes from *A. thaliana* allowed us to define an optimal combination of three genes, *DXS*, *HDR* and *GGPPS*, for production of casbene in *N. benthamiana*. Our finding that overexpression of *HDR* rescues the dominant negative effect on casbene production of expression of the preceding enzyme in the MEP pathway, HDS, mirrors what was found in *E. coli* where it is understood that the HDR enzyme equivalent removes the cytotoxic intermediate and product of HDS, HMBPP (Li *et al*., 2017). The very positive effect of overexpression of AtHDR on casbene production could therefore be due to reduction in HMBPP levels instead of or in addition to the HDR step being rate‐limiting. Transient expression also allowed us to select an optimal set of gene promoters for casbene production and, interestingly, we found that promoters that drive expression at intermediate rather than high levels (as determined by GFP fluorescence) were most effective at increasing flux to casbene through the MEP pathway. Placing the chosen MEP pathway gene constructs in a single vector also proved to increase casbene production compared to transient expression of casbene synthase alone. The resulting vector, carrying the optimal set of MEP pathway genes, was then used for stable transformation of *N. benthamiana*. The ability to up‐regulate the MEP pathway and direct flux to casbene was demonstrated to be essential when it comes to engineering production of casbene‐derived diterpenoids such as jolkinol C and *epi*‐jolkinol C in stable transgenic lines of *N. benthamiana*, highlighting the importance of optimizing substrate supply in metabolic engineering of complex diterpenoids in stable production platforms. Such platforms may prove valuable for production of bioactive diterpenoids but the pathways for production of many of these remain to be fully elucidated and will involve a process of step‐by step gene discovery. We demonstrate that an *N. benthamiana* line engineered to produce jolkinol C and *epi*‐jolkinol C can be a valuable tool when used in combination with transient expression for candidate gene function determination. This approach was used to reveal the function of the enzyme responsible for the double bond reduction at the C12‐C13 position on jolkinol C and epi‐jolkinol C, which could be an important step in the biosynthesis of more complex diterpenes including ingenol mebutate and tigilanol tiglate. Our discovery could therefore contribute to engineering the production of medicinal compounds in heterologous systems.

## Material and methods

### Transient expression of genes in *Nicotiana benthamiana*

cDNAs from *Arabidopsis thaliana* plastidial MEP genes and plastidial *GGPPS11* (Beck *et al*., 2013) were prepared from total RNA samples using Superscript III reverse transcriptase (Invitrogen) and random hexamer primers. Accession numbers and references for these genes can be found in Table S1 (Phillips *et al*., 2008, 2008,2008, 2008; Ruiz‐Sola *et al*., 2016). The open reading frames (ORFs) of these genes were subsequently amplified with Phusion Pfu polymerase (New England Biolabs) using primers designed on NEBuilder for Gibson assembly and detailed in Table S1. Casbene synthase, cytochrome P450 genes *CYP726A20* and *CYP71D495* and the four alkenal reductases from *Jatropha curcas* have been amplified in previous work (King *et al*., 2014) and were already available in pEAQ‐HT vectors (Sainsbury *et al*., 2009). ORFs from *Arabidopsis* genes were cloned with NEB Gibson Assembly Mastermix according to the manufacturer’s protocol in pEAQ‐HT vector, allowing each gene to be positioned under the control of an improved cauliflower mosaic virus (CAMV) 35S promoter (Sainsbury and Lomonossoff, 2008).

For assembly with Modular Cloning (MoClo), ORFs were first domesticated, *that is* removal of the restriction sites BsaI and BpiI when necessary. The domesticated coding sequences (CDS) were cloned in level −1 and level 0 vectors using MoClo Tool Kit (Addgene) (Weber *et al*., 2011; Werner *et al*., 2012), following the long protocol described by the manufacturer. CDS were then combined to different promoters and terminators provided by the MoClo Plant Parts kit (Addgene) (Engler *et al*., 2014) in the level 1 vector. Genes coding for *NptII* (kanamycin resistance cassette) and *Bar* (bialaphos/glufosinate/Basta resistance cassette) were also available in the Plant Parts kit. The genes obtained were finally assembled in the level 2 vector intended for transient and stable expression (see Table S3 for details on the transcriptional units).

The expression vectors were then transformed into *Agrobacterium tumefaciens* LBA4404 using the freeze thaw method (Höfgen and Willmitzer, 1988). A pEAQ‐HT vector with eGFP was also created in previous work (King *et al*., 2016) to visualize and delimitate the infiltrated areas of the leaves when co‐transformed with the candidate genes. *A. tumefaciens* cultures were initially grown to an OD between 2 and 3 in YEB media (5 g/L beef extract, 1 g/L yeast extract, 5 g/L peptone, 5 g/L sucrose and 0.5 g/L MgCl_2_), pelleted at 4000 *g* for 15 min and resuspended in pre‐infiltration media (10 mM MgCl_2_, 200 μM acetosyringone and 0.015%) at an OD of 5 to be left for 1–2 h. For mixed infiltrations, the same amount of cells were added such that each strain was present at the same density of 0.2 OD in the final infiltration volume. Vacuum infiltration of *N. benthamiana* plants was performed by dipping plants into the infiltration media (10 mM MgCl_2_, 200 μM acetosyringone, 0.015% Silwet L‐77 plus *A. tumefaciens*) in a degassing chamber at 50 mBar for 60 s. Five days after *Agrobacterium* infiltration, leaves showing GFP signal under UV were harvested, freeze‐dried and ground for 30 s with a steel bead at 30 Hz in a Retsch homogenizer in order to perform metabolomic extraction.

### Isolation and quantification of diterpenoids

To detect and quantify the production of casbene in transiently expressed plants, around 200 mg of dry material were extracted with 5 mL of hexane containing 100 µg/mL of β‐caryophyllene then sonicated for 15 min. 100 µL of the extracts were used for GC‐MS analysis and 2 µL were injected in a Leco Pegasus IV GC‐TOF instrument. The GC oven was fitted with a Restek RTX‐5SIL MS capillary column (30m, 0.25‐mmID, 0.25 mm *df*). The oven temperature was set at 100°C for 2 min and then increased to 300°C at a rate of 5°C min^−1^. Mass spectral data were acquired over the m/z range of 50 to 450 in positive electron ionization mode at −70 eV.

For the quantification of casbene and jolkinols in stable and transiently transformed plants, *ca*. 250 mg of dry material were extracted with 1 mL of ethyl acetate containing 10 µg/mL of β‐caryophyllene and 20 µg/mL of phorbol myristate acetate (PMA). After an overnight shaking at 2200 *g* on a IKA Vibrax VXR basic shaker, the samples were centrifuged and 100 µL of the supernatant was used directly for GC‐MS, while the rest was evaporated in a GeneVac EZ‐2 plus and resuspended in 250 µL of methanol for UPLC‐MS analysis. A 2 µL aliquot was analysed in the Waters Acquity™ UPLC using an Acquity UPLC^®^ BEH C18 column (Waters, 1.7 µm, 2.1 × 100 mm) kept at 60°C. Mobile phases A and B were water with 5% methanol + 0.1 % formic acid; and methanol + 0.1 % formic acid, respectively. A flow rate of 0.5 ml/min was used. The gradient profile was as follows: 30 s at 40% B; a linear gradient lasting 25 min from 40% B to 100% B, then held for 5 min; and a final step of 40% B maintained for 2 min. Mass spectral data were acquired over the m/z range of 100–1000 in positive polarity mode using an APCI source.

Casbene was quantified by determination of the total ion chromatogram (TIC) peak area and comparison to the peak area of the internal standard, β‐caryophyllene. Jolkinol C, *epi*‐jolkinol C, 6‐hydroxy‐5‐ketocasbene, 6,9‐dihydroxy‐5‐ketocasbene, 12,13‐dihydro‐jolkinol C and 12,13‐dihydro‐*epi*‐jolkinol C were quantified and/or detected by determination of their main ion base peak area (m/z 299 for jolkinol C and epi‐jolkinol C; m/z 285 or 303 for 6‐hydroxy‐5‐ketocasbene; m/z 301 or 319 for 6,9‐dihydroxy‐5‐ketocasbene, 12,13‐dihydro‐jolkinol C and 12,13‐*epi*‐dihydro‐jolkinol C) and comparison with the main ion base peak area of the internal standard PMA (m/z 389).

### Accumulation and purification of compounds for NMR spectroscopy

For 6,9‐dihydroxy‐5‐ketocasbene, we vacuum infiltrated 48 N*. benthamiana* WT plants with *Agrobacterium* strains containing plasmids for overexpression of precursor genes, *JcCAS*, *JcCYP726A20* and *JcCYP71D495* to obtain 6.1 g of freeze‐dried material. This material was extracted with 15 volumes of ethyl acetate during 5 days on a gentle rotary shaker. The extract was dried on a rotary evaporator to yield 480 mg of green oily residue, resuspended in 10 mL of hexane/ethyl acetate (70:30, v/v) and subjected to one round of flash chromatography on a PuriFlashⓇ 4250 system (Interchim). We used a 40 g Bucchi silica column and a hexane/ethyl acetate gradient as described in the King *et al*., 2014 to fractionate the extract into 80 samples. Fractions were analysed by UPLC‐MS and those containing 6,9‐dihydroxy‐5‐ketocasbene were combined and dried to yield 0.23 mg of compound. This was sufficiently pure to allow an 1H NMR analysis to be recorded in CDCl_3_ with a Bruker AVIII 700 MHz spectrometer instrument.

For 12,13‐dihydro‐jolkinol C and 12,13‐dihydro‐epi‐jolkinol C, we applied the same procedure described above. We used WT tobacco to over‐express the same genes and the alkenal reductase 3 or double‐bond reductase *DBR*. We infiltrated 96 plants to obtain 21 g of freeze‐dried material leading to 1.15 g of green oily residue. The fractions obtained from the flash chromatography allowed accumulation of 0.23 mg and 0.14 mg of the metabolites later identified as 12,13‐dihydro‐jolkinol C and 12,13‐dihydro‐epi‐jolkinol C, respectively.

### Stable transformation of *Nicotiana benthamiana*

*Nicotiana benthamiana* stable transformation was performed following the leaf discs method (Horsch *et al*., 1989). Leaves from 6‐week‐old *N. benthamiana* were first sterilized in 10% bleach for 10 min and then rinsed 4–5 times in sterile distilled water (Clemente, 2006). Discs were cut with a sterile cork borer of 1 cm diameter and soaked in an *Agrobacterium* co‐cultivation solution consisting of 4.3 g/L of MS medium M0221 (Duchefa), 30 g/L of anhydride glucose, 100 mg/L of myo‐inositol, 0.5 mg/L of the vitamins nicotinic acid, thiamine‐HCl and pyridoxine, 2 mg/L of glycine and few drops of KOH 1N to adjust the pH to 5.7–5.8. *Agrobacterium* LBA4404 (Hoekema *et al*., 1983) containing vectors of interest were grown in preliminary culture for 2 days in YEB media then centrifuged and washed with 1 mL of 10mM MgSO_4_ before being resuspended in 1 mL of the co‐cultivation solution described above. Leaf discs infected with *Agrobacterium* were then dried on sterile blotting paper and incubated for 3–4 days onto solid co‐cultivation medium containing 0.1 mg/L of 1‐Naphthaleneacetic acid (NAA) and 1 mg/L of 6‐Benzylaminopurine (BAP). After this incubation period, discs were transferred on the same medium supplemented with the plant selection agent (100 mg/L for kanamycin and 5 mg/L for glufosinate) and 500 mg/L of cefotaxime to eliminate the bacteria. Discs were transferred every 2–3 weeks onto fresh co‐cultivation plates to promote and improve appearance of buds and calli. Shoots started to appear 30–40 days after transformation and were transplanted into sterile pots containing rooting medium (2.65 g/L of modified MS n°4 M0238 from Duchefa, 825 mg/L of NH_4_NO_3_, 30 g/L of sucrose, 100 mg/L of myo‐inositol, 0.5 mg/L of the same 3 vitamins as the co‐cultivation medium, a few drops of KOH to adjust the pH at 5.7–5.8 and 6 g/L of agar). The doses of kanamycin and glufosinate were doubled in this medium to eliminate the non‐transformed explants. Roots started developing 15–30 days after transfer, allowing the transformed seedlings to be put into soil. Primary transformants were tested by PCR and on their metabolic content to confirm the success of the transformation.

Seeds of the subsequent generations were sown on a germination medium (4.4 g/L MS medium M0221 from Duchefa, 10 g/L sucrose, 100 mg/L myo‐inositol, 0.5 mg/L nicotinic acid and pyridoxine, 1 mg/L thiamine, few drops of KOH to adjust at pH 5.7–5.8 and 6 g/L agar) containing 500 mg/L kanamycin and 10 mg/L glufosinate to perform segregation tests and estimate the copy number of each transgene.

Following germination and appropriate selection, WT and transgenic seedlings were transferred to F2 + S seed and modular compost (Levingston Advance) and cultivated in a growth chamber under white fluorescent lamps set at 22°C during the day (16 h) and 20°C during the night (8 h).

## Conflict of interest

The authors declare that they have no conflict of interest.

## Author contributions

EF designed experiments, performed experiments and analysed data. TC, AD, AC, AK and GD performed experiments and analysed data. EF and IG wrote the manuscript. IG contributed to the conception, design and analysis of the study. All authors read and approved the manuscript.

## Supporting information

**Figure S1** Casbene content in *N. benthamiana* when co‐infiltrated with candidate genes plus *AtHDS*.
**Figure S2** Co‐expression of the jolkinol pathway genes compared to the expression of the multigene constructs.
**Figure S3** Strategy for generation of stable transformants.
**Figure S4** NMR data for 6,9‐dihydroxy‐5‐ketocasbene.
**Figure S5** Morphological difference of *N. benthamiana* WT and co‐transformants at different generations.
**Figure S6** Casbene derivatives content in the T2 homozygous populations from the three independent primary co‐transformants.
**Figure S7** Diterpenoid biosynthesis gene cluster identified in *J. curcas* genome by King et al. (2016).
**Figure S8** NMR data for 12,13‐dihydro‐Jolkinol C and 12,13‐dihydro‐*epi*‐Jolkinol C.

**Table S1** CDS cloned from *Arabidopsis thaliana* and Gibson primers used to amplify them.
**Table S2** List of promoters used in this work and classification in relation to their strength.
**Table S3** List of MoClo components and modules used in this work. Design of level 1 and level 2 constructs.
