# Supplementary material for: Developing a Nicotiana benthamiana transgenic platform for high‐value diterpene production and candidate gene evaluation
Source: Plant Biotechnol J. 2021 Mar 18;19(8):1614–23. doi: 10.1111/pbi.13574 (PMC8384591; doi:10.1111/pbi.13574)

**Figure S1 Casbene content in *N. benthamiana* when co-infiltrated with candidate genes plus *AtHDS*.** Yellow: expression of *JcCAS* *vs* *JcCAS* + *AtHDS*. Green: co-expression of *AtDXS* + *JcCAS* *vs* the same combination + *AtHDS*. Pink: co-expression of *AtDXS* + *AtGGPPS* + *JcCAS* *vs* the same combination + *AtHDS*. Blue: co-expression of *AtDXS* + *AtHDR* + *AtGGPPS* + *JcCAS* *vs* the same combination + *AtHDS*.

Average casbene content (in µg/mg DW) ± standard deviations are displayed (n = 3). Distinct symbols indicate significant differences between the quantitative values (P < 0.05, F-test and T-test). Each colour represents an unrelated statistical group.


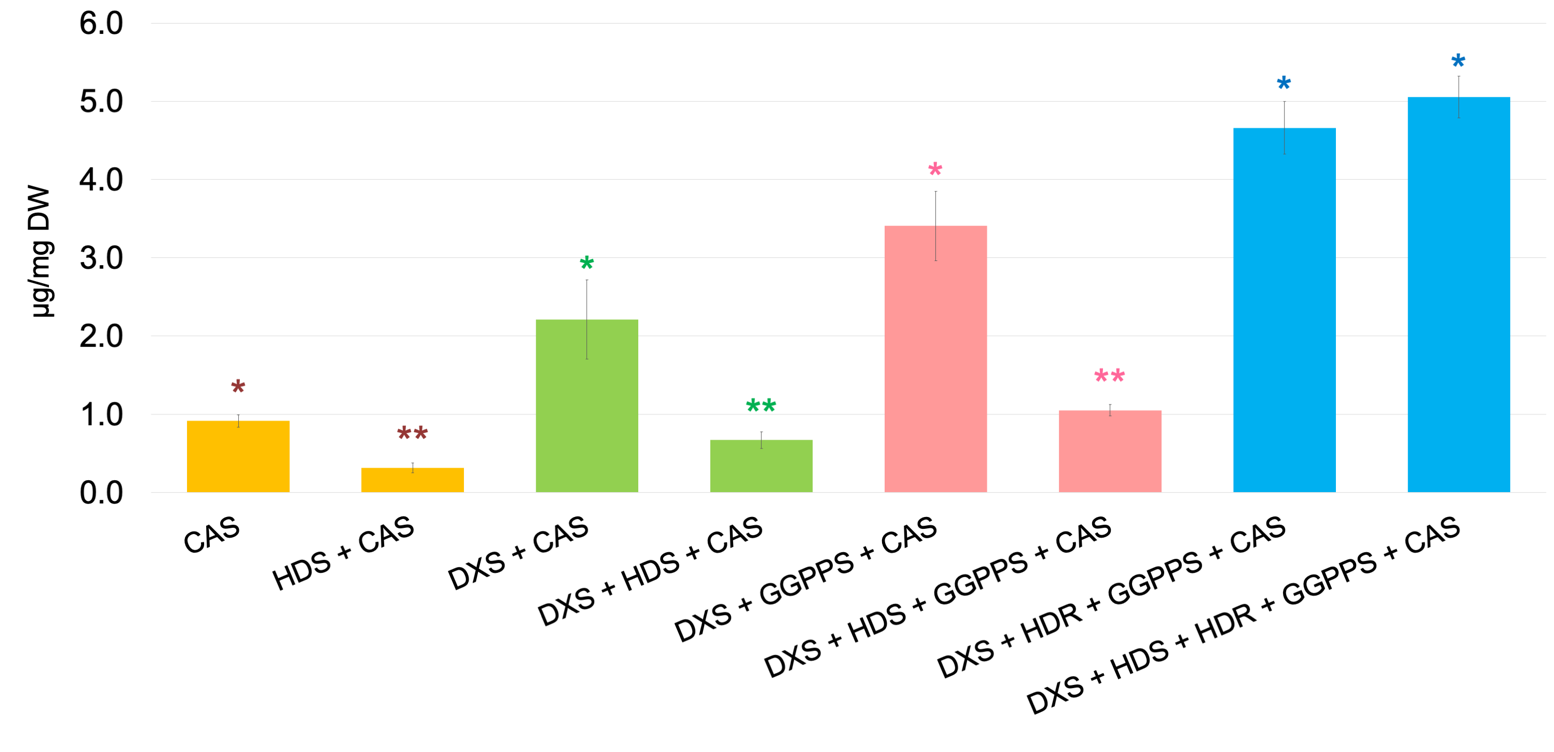


**Figure S2 Co-expression of the jolkinol pathway genes compared to the expression of the multigene constructs.** (a) Multigene constructs obtained by MoClo and their designation. “A-CP” and “B-CP” refer to multigene constructs carrying *JcCAS*, *JcCYP726A20* and *JcCYP71D495* driven by group A and B of promoters, respectively. Both multigene constructs also carry the *NptII* gene in position 4, conferring resistance to the herbicide kanamycin. *NptII* gene was included in the MoClo kit. (b) Content of casbene (green), *epi*-jolkinol C (blue) and jolkinol C (yellow) in leaves of *N. benthamiana* agro-infiltrated with single candidate genes vs multigene constructs (in ng/mg DW).

Average content of each compound ± standard deviations are displayed (n = 3). The different symbols indicate there is a significant difference between the quantitative values (P < 0.05, F-test and T-test). Each color represents an unrelated statistical group. Casbene content measured by GC-MS. Jolkinol C and *epi*-jolkinol C contents measured by UPLC-MS.


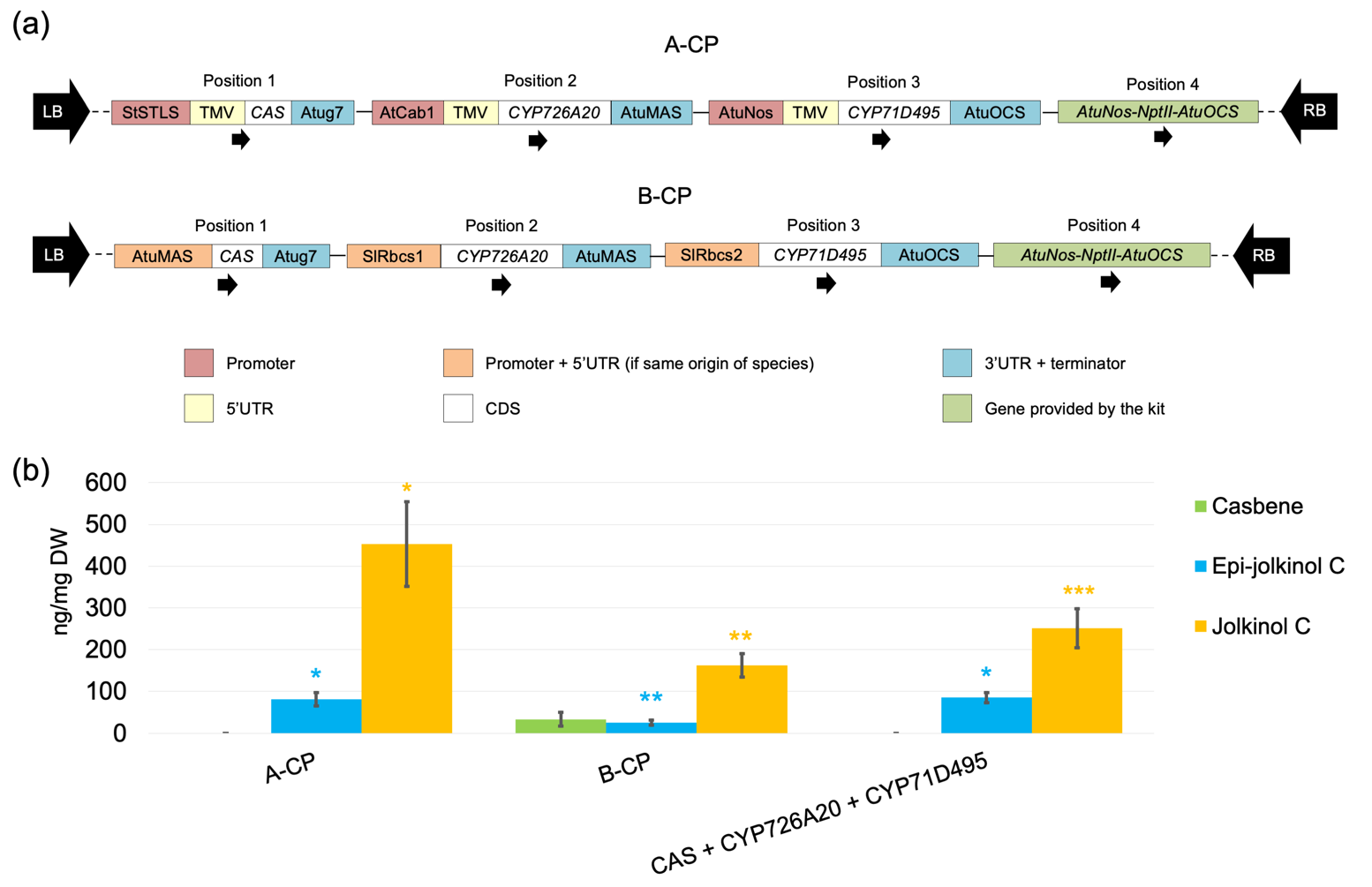


**Figure S3 Strategy for generation of stable transformants.** 18 primary transformants coming from the single transformation have been obtained. The co-transformation allowed us to acquire 29 T0 lines (including 2 controls) shared unevenly between the 5 co-transformation conditions.

**
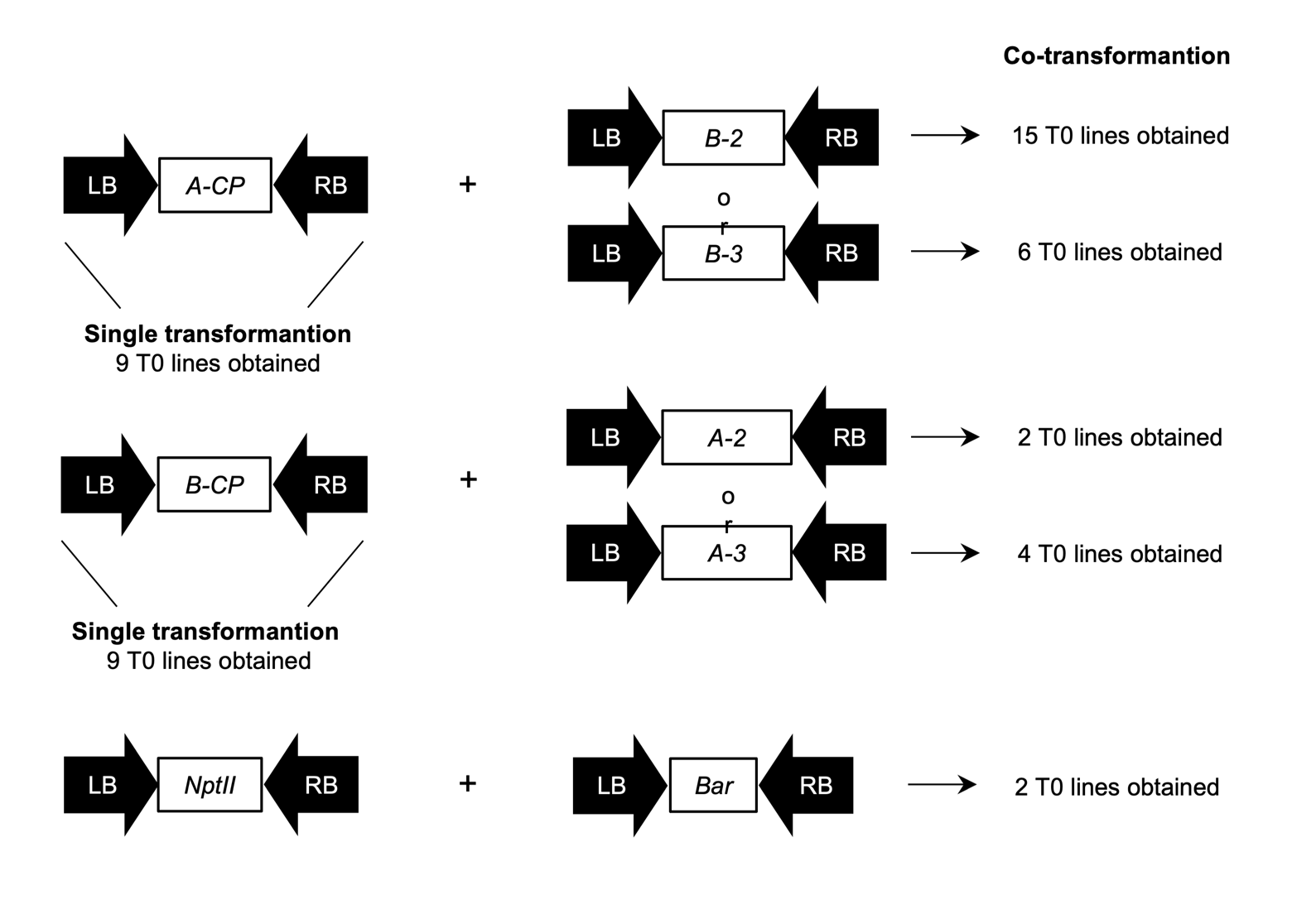
**

**Figure S4 NMR data for 6,9-dihydroxy-5-ketocasbene.**

Data for 6,9-dihydroxy-5-ketocasbene:^1^H NMR (700 MHz, CDCl_3_): δ 6.29 (d, J = 10 Hz, 1H (H-3)), 5.27 (d, J = 9 Hz, 1H (H-6)), 5.20 (d, J = 9 Hz, 1H (H-7)), 4.70 (br d, J = 10 Hz, 1H, (H-11)), 4.15 (dd, J = 10, 6 Hz, 1H, (H-9)), 2.33 (m, 1H (H-10)), 2.31 (dd, J = 14, 10 Hz, 1H (H-10)), 2.25 (m, 1H, (H-13)), 2.11 (m, (H-14)), 1.95 (d, J = 1 Hz, 3H, (H-18)) 1.74 (m, 1H (H-13)), 1.71 (d, J = 1 Hz, 3H (H-19)), 1.61 (s, 3H (H-20)), 1.56 (dd, J = 10,8 Hz, 1H (H-2)), 1.22 (ddd, J = 12, 8, 2 Hz, 1H (H-1)), 1.18 (s, 3H (H-16)), 1.03 (s, 3H (H-17)), 0.80 (m, 1H (H-14));^13^C NMR (175 MHz, CDCl_3_): δ 199.6 (C-5), 145.2 (C-3), 143.6 (C-8), 137.6 (C-12), 134.5 (C-4), 125.6 (C-7), 119.4 (C-11), 77.7 (C-9), 67.7 (C-6), 39.8 (C-13), 35.7 (C-1), 31.6 (C-10), 29.2 (C-16), 28.0 (C-2), 27.7 (C-15), 25.4 (C-14), 16.0 (C-17), 15.4 (C-20), 12.0 (C-18), 10.2 (C-19).

^1^H NMR (700 MHz)


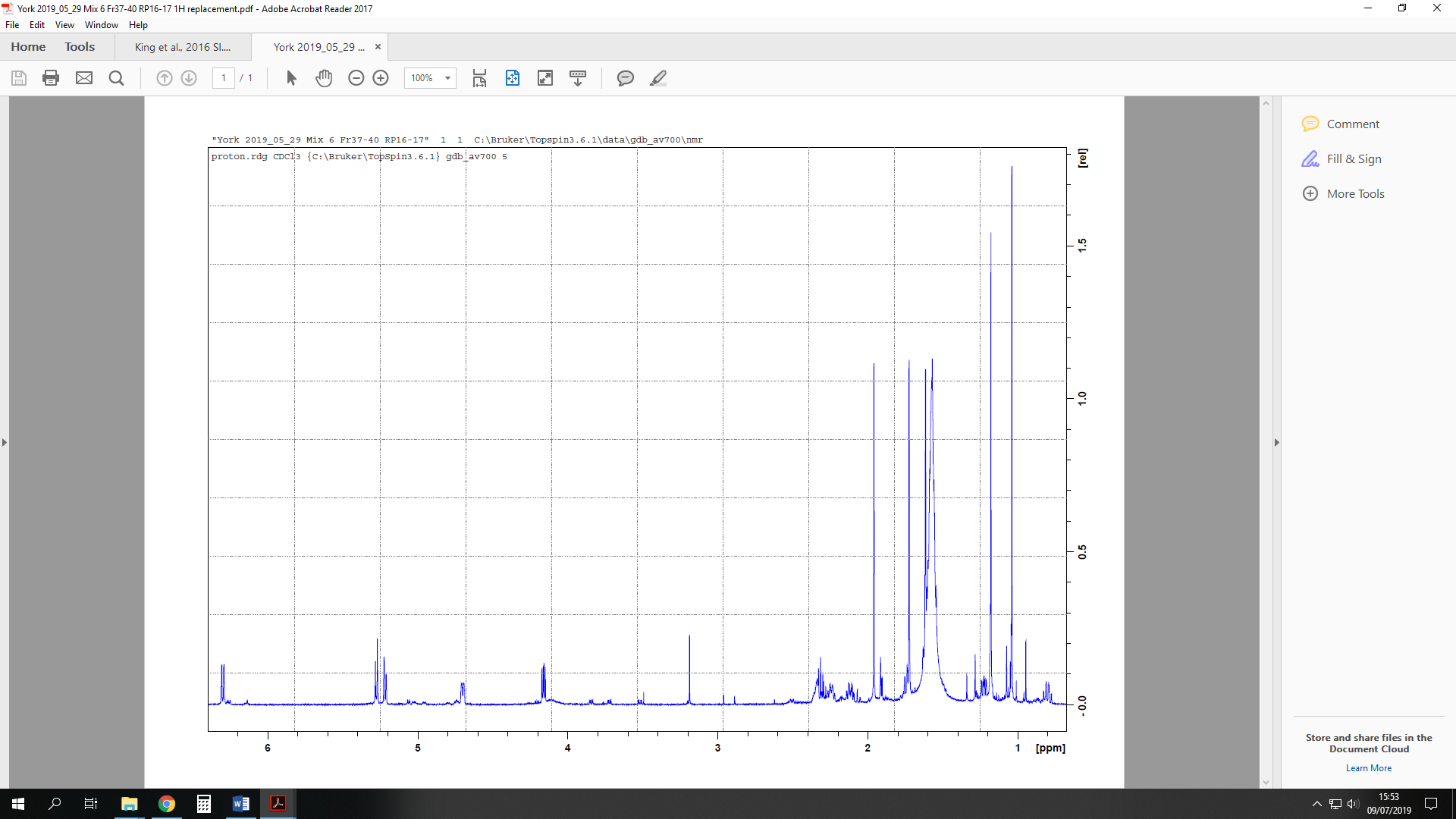


^13^C NMR (175 MHz)


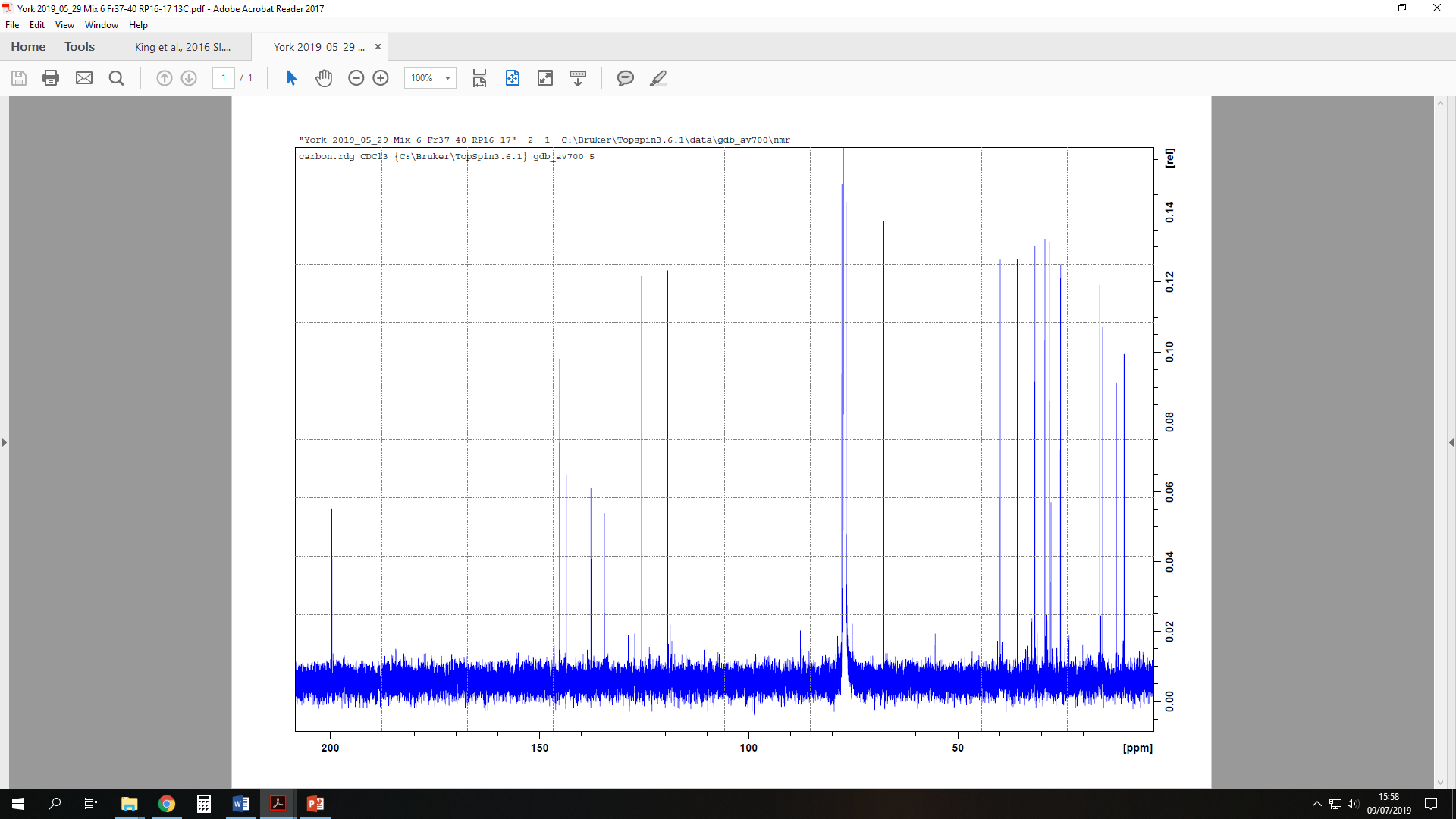


Edited-HSQC


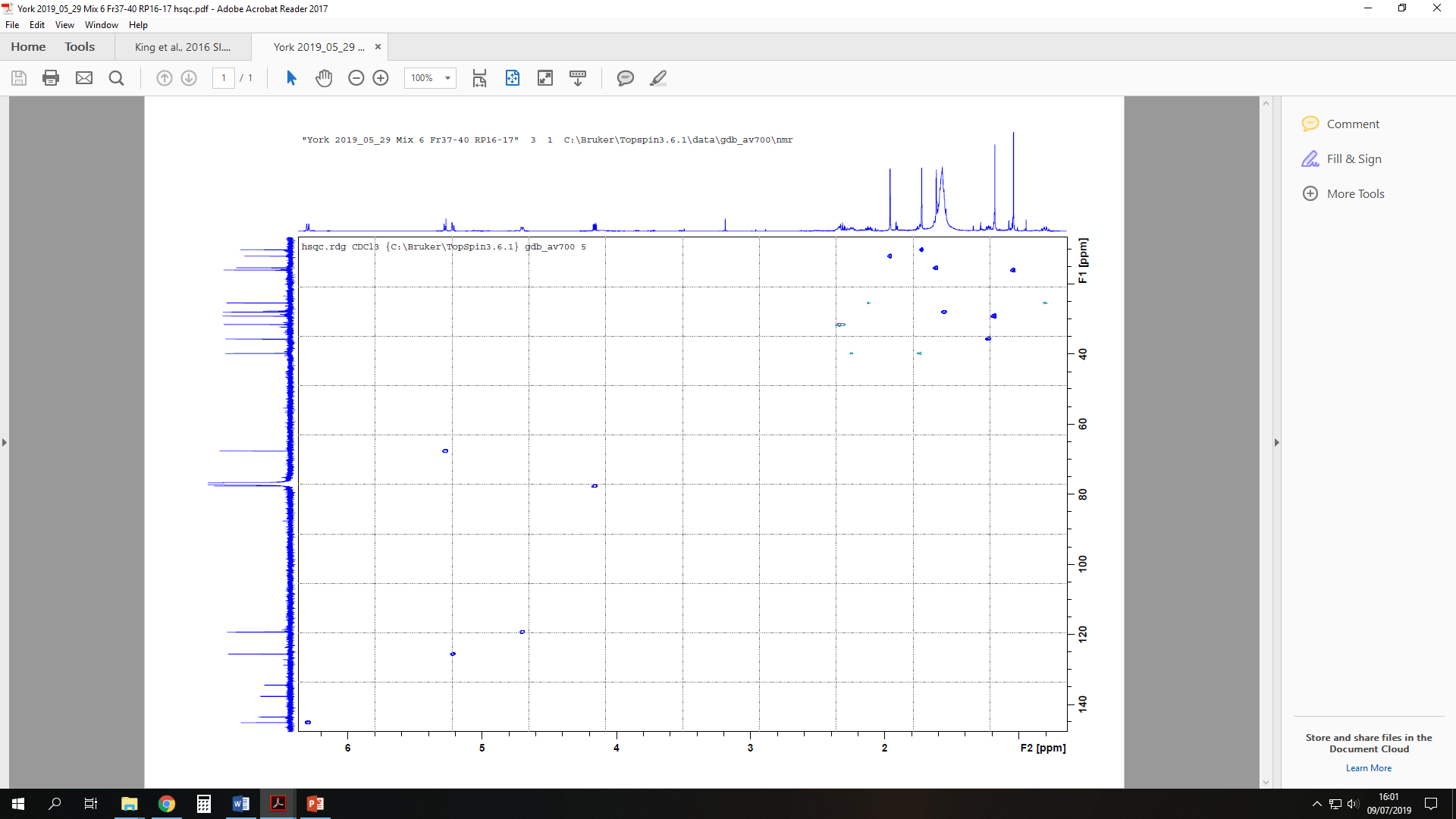


**Figure S5 Morphological difference of *N. benthamiana* WT and co-transformants at different generations.** (a) T0 generation: difference of *N. benthamiana* pods between WT and co-transformant B-2/A-CP n°10. (b) T3 generation: general shape difference between WT tobacco and T3 lines NbJolk-C descending from B-2/A-CP n°4.

† *N. benthamiana* WT was generated through the process of leaf disc and tissue culture.


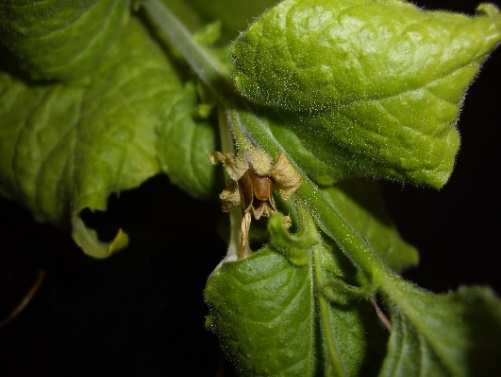

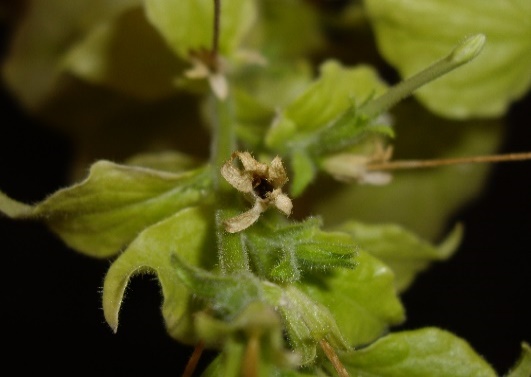


WT ^†^

B-2/A-CP n°10

(a)


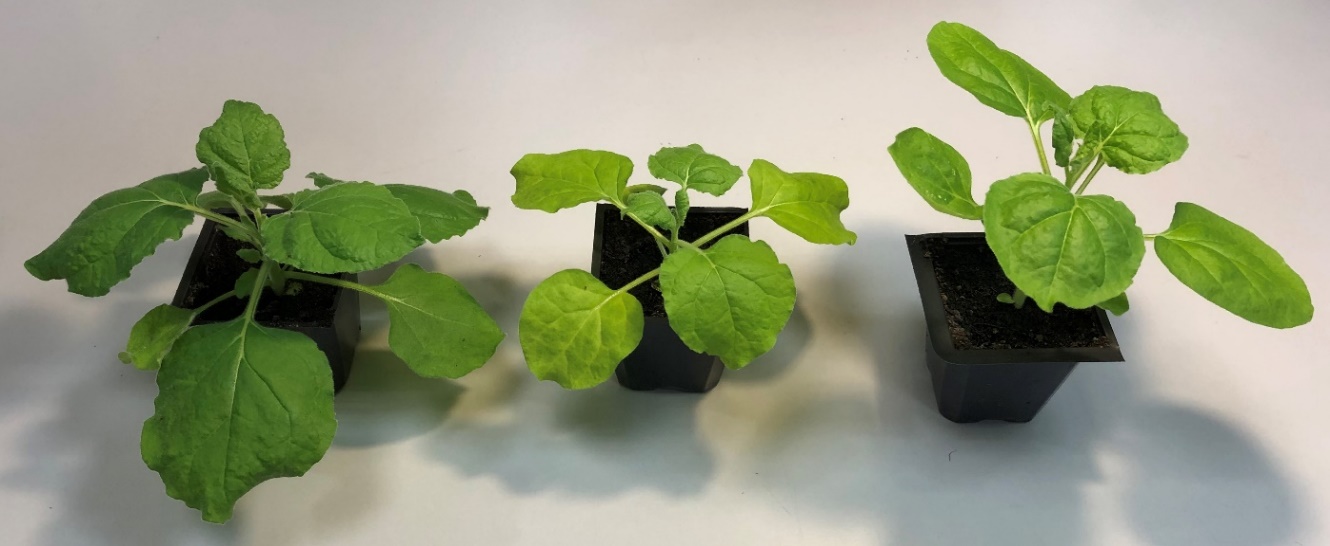

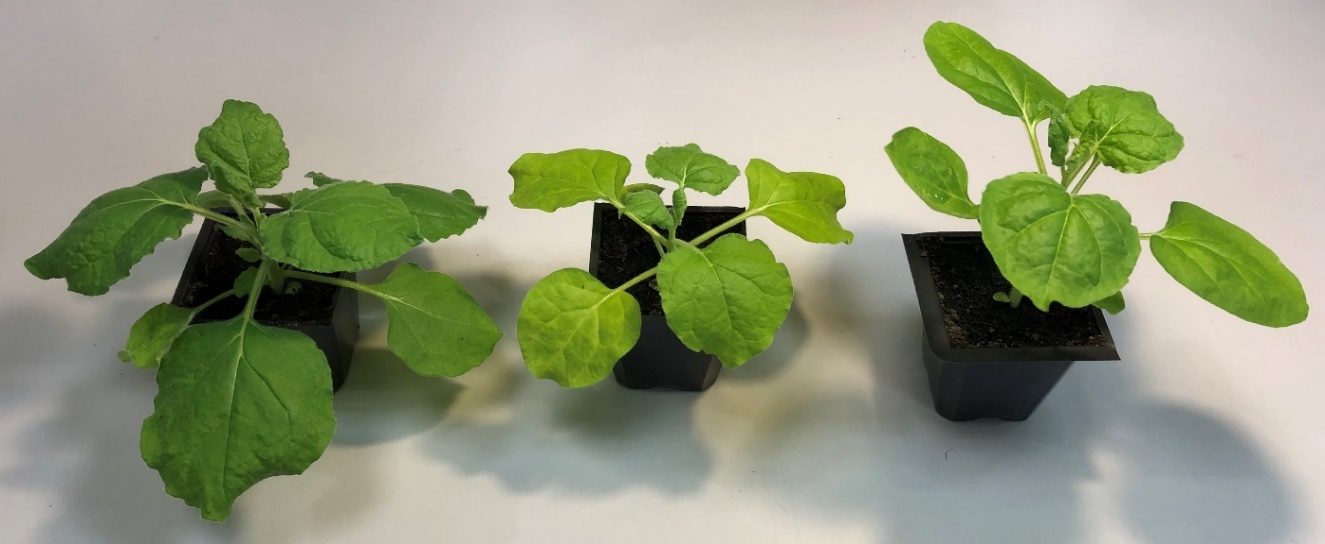


T3 NbJolk-C

WT ^†^

(b)

**Figure S6 Casbene derivatives content in the T2 homozygous populations from the three independent primary co-transformants**. Average content of each compound ± standard deviations are displayed (n = 3).

**Figure S7 Diterpenoid biosynthesis gene cluster identified in *J. curcas* genome by King *et al*., 2016.** The different classes of enzymes have been colour-coded, e.g., alkenal reductases are shown in orange.

**Figure S8 NMR data for 12,13-dihydro-Jolkinol C and 12,13-dihydro-*epi*-Jolkinol C.**

Data for 12,13-Dihydro-jolkinol C:^1^H NMR (700 MHz, CDCl_3_): δ 5.16 (d, J = 10 Hz, 1H (H-5)), 3.98 (d, J = 10 Hz, 1H (H-4)), 3.57 (dqd, J = 7,7,3 Hz, 1H (H-13)), 2.63 (m, 1H, (H-1)), 2.62 (m, 1H, (H-2)), 2.52 (dd, J = 13, 7 Hz, 1H (H-7)), 1.83 (ddd, J = 13, 13, 5 Hz, 1H (H-7)), 1.75 (m, 1H, (H-1)), 1.72 (s, 3H (H-17)), 1.72 (m, 1H (H-8)), 1.59 (m, 1H (H-12)), 1.42 (m, 1H (H-12)) 1.27 (d, J = 7 Hz, 3H (H-16)), 1.11 (d, J = 7 Hz, 3H (H-20)), 1.00 (s, 3H (H-18)), 0.97 (m, 1H (H-8)), 0.68 (s, 3H (H-19)), 0.29 (m, 1H, (H-11)), 0.28 (m, 1H, (H-9));^13^C NMR (175 MHz, CDCl_3_): δ 217.4 (C-3), 217.2 (C-14), 146.4 (C-6), 113.5 (C-5), 88.7 (C-15), 58.9 (C-4), 41.6 (C-1), 39.1 (C-2), 38.3 (C-13), 37.5 (C-7), 28.6 (C-18), 26.7 (C-9), 24.7 (C-12), 24.5 (C-8), 21.8 (C-17), 18.4 (C-11), 18.0 (C-16), 17.0 (C-10), 15.4 (C-19), 13.7 (C-208).

Data for 12,13-Dihydro-*epi*-jolkinol C:^1^H NMR (700 MHz, CDCl_3_): δ 5.13 (d, J = 10 Hz, 1H (H-5)), 3.82 (d, J = 10 Hz, 1H (H-4)), 3.58 (dqd, J = 7,7,2 Hz, 1H (H-13)), 2.55 (m, 1H, (H-2)), 2.51 (dd, J = 12, 6 Hz, 1H, (H-7)), 2.24 (dd, J = 13, 8 Hz, 1H (H-1)), 1.96 (dd, J = 13, 11 Hz, 1H (H-1)), 1.84 (m, 1H, (H-6), 1.72 (d, J = 1 Hz, 3H (H-17)), 1.72 (m, 1H (H-8)), 1.65 (m, 1H (H-12)), 1.43 (m, 1H (H-12)) 1.22 (d, J = 7 Hz, 3H (H-16)), 1.11 (d, J = 7 Hz, 3H (H-16)), 1.00 (s, 3H (H-18)), 0.97 (m, 1H (H-8)), 0.69 (s, 3H (H-19)), 0.29 (m, 1H, (H-11)), 0.28 (m, 1H, (H-9);^13^C NMR (175 MHz, CDCl_3_): δ 217.5 (C-3), 216.7 (C-14), 146.3 (C-6), 113.8 (C-5), 87.7 (C-15), 58.3 (C-4), 42.3 (C-1), 40.7 (C-2), 38.5 (C-13), 37.5 (C-7), 28.6 (C-18), 26.6 (C-9), 24.7 (C-12), 24.4 (C-8), 21.7 (C-17), 18.4 (C-11), 16.6 (C-10), 15.7 (C-16), 15.4 (C-19), 13.7 (C-20).

Edited-HSQC 12,13-Dihydro-jolkinol C


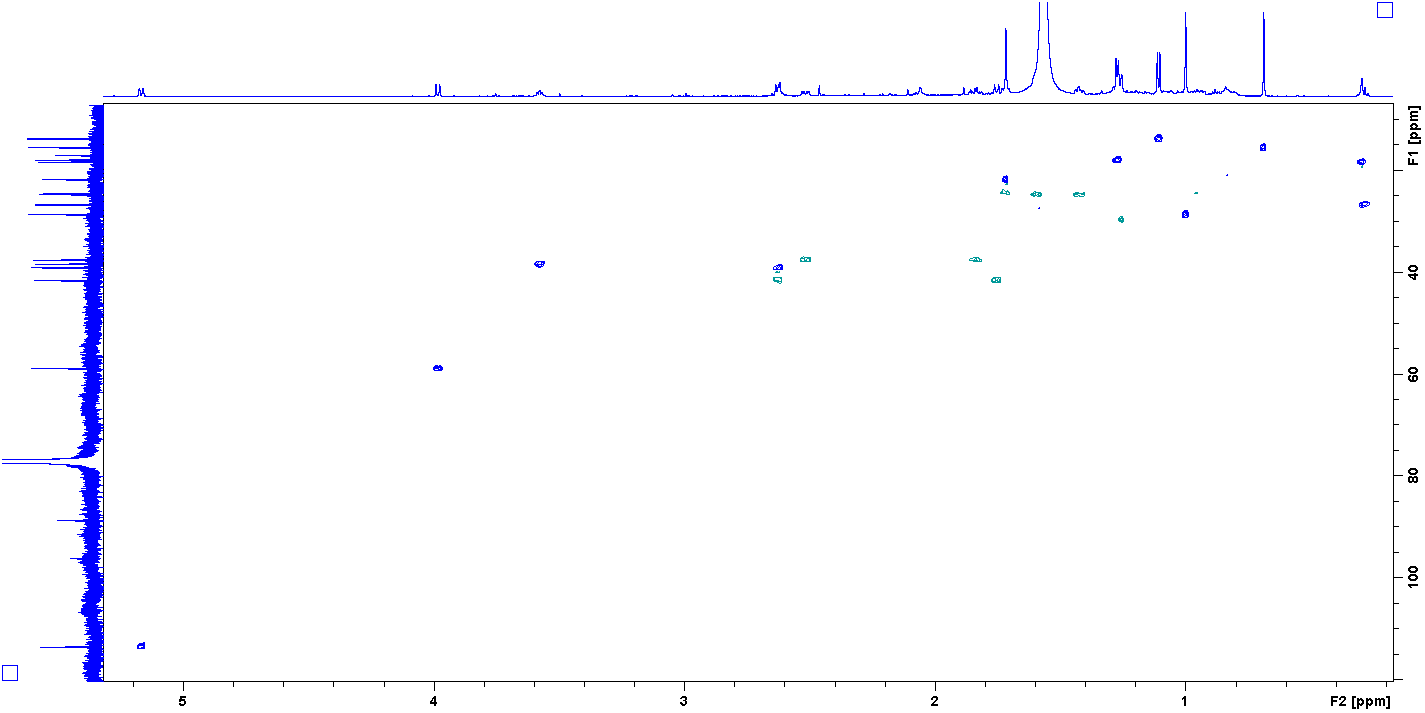


^1^H NMR (700 MHz) 12,13-Dihydro-*epi*-jolkinol C


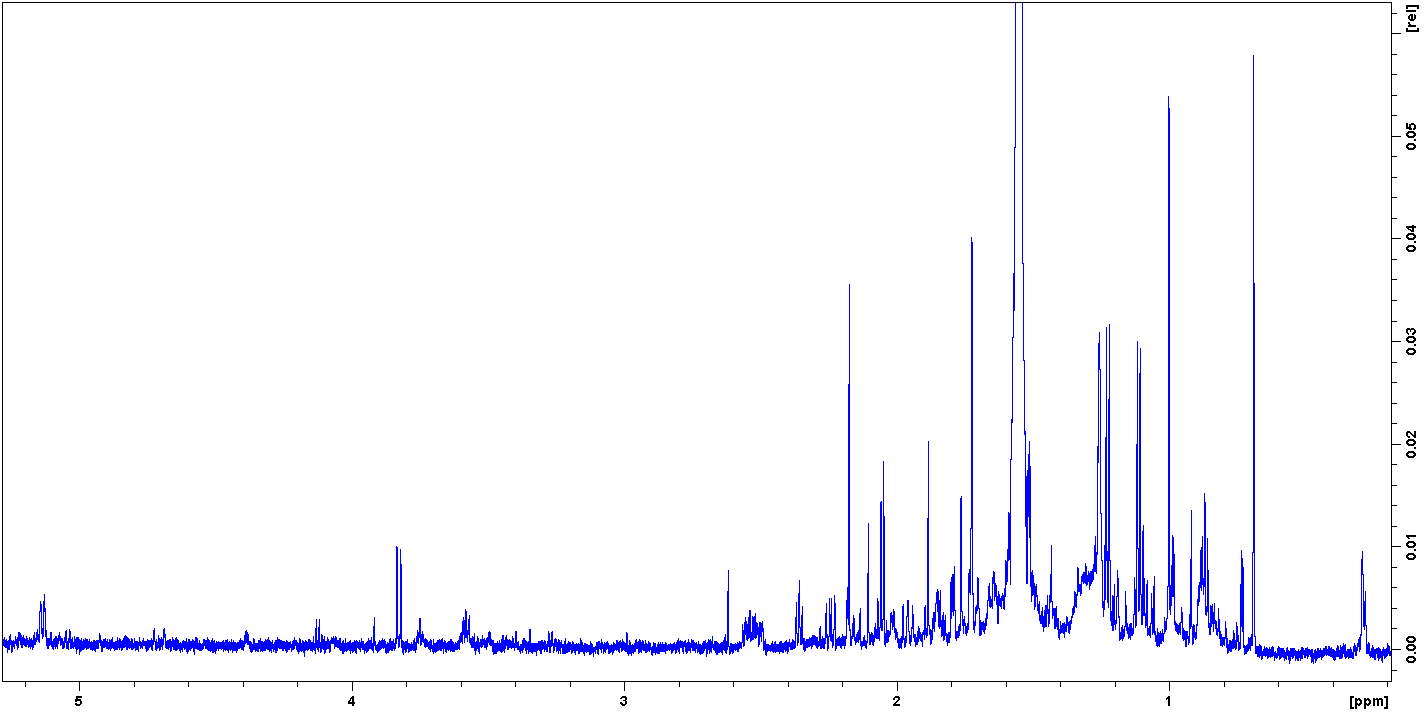


Edited-HSQC 12,13-Dihydro-*epi*-jolkinol C


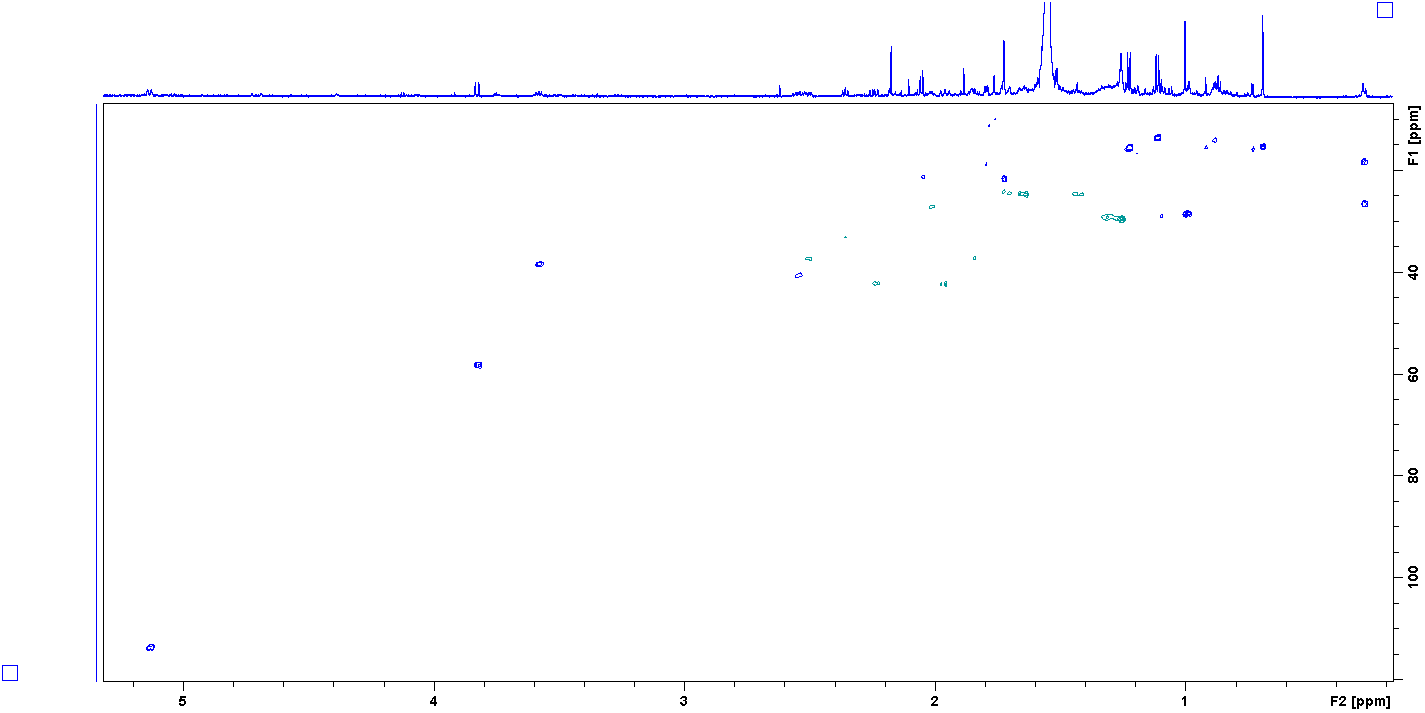

Supplement: Supplementary file 1 — Figure S1 Casbene content in N. benthamiana when co‐infiltrated with candidate genes plus AtHDS. Figure S2 Co‐expression of the jolkinol pathway genes compared to the expression of the multigene constructs. Figure S3 Strategy for generation of stable transformants. Figure S4 NMR data for 6,9‐dihydroxy‐5‐ketocasbene. Figure S5 Morphological difference of N. benthamiana WT and co‐transformants at different generations. Figure S6 Casbene derivatives content in the T2 homozygous populations from the three independent primary co‐transformants. Figure S7 Diterpenoid biosynthesis gene cluster identified in J. curcas genome by King et al. (2016). Figure S8 NMR data for 12,13‐dihydro‐Jolkinol C and 12,13‐dihydro‐epi‐Jolkinol C. [file PBI-19-1614-s002.docx]
